# *Drosophila*, a powerful model to study virus-host interactions and pathogenicity in the fight against SARS-CoV-2

**DOI:** 10.1186/s13578-021-00621-5

**Published:** 2021-06-13

**Authors:** Joyce van de Leemput, Zhe Han

**Affiliations:** 1 Center for Precision Disease Modeling, Department of Medicine, University of Maryland School of Medicine, Baltimore, MD 21201 USA; 2 Division of Endocrinology, Diabetes and Nutrition, Department of Medicine, University of Maryland School of Medicine, Baltimore, MD 21201 USA

**Keywords:** *Drosophila*, Coronavirus, SARS-CoV-2, Animal models, Virus-host interactions, Primary determinants of pathogenicity

## Abstract

The COVID-19 pandemic is having a tremendous impact on humanity. Although COVID-19 vaccines are showing promising results, they are not 100% effective and resistant mutant SARS-CoV-2 strains are on the rise. To successfully fight against SARS-CoV-2 and prepare for future coronavirus outbreaks, it is essential to understand SARS-CoV-2 protein functions, their host interactions, and how these processes convey pathogenicity at host tissue, organ and systemic levels. In vitro models are valuable but lack the physiological context of a whole organism. Current animal models for SARS-CoV-2 research are exclusively mammals, with the intrinsic limitations of long reproduction times, few progeny, ethical concerns and high maintenance costs. These limitations make them unsuitable for rapid functional investigations of virus proteins as well as genetic and pharmacological screens. Remarkably, 90% of the SARS-CoV-2 virus-host interacting proteins are conserved between *Drosophila* and humans. As a well-established model system for studying human diseases, the fruit fly offers a highly complementary alternative to current mammalian models for SARS-CoV-2 research, from investigating virus protein function to developing targeted drugs. Herein, we review *Drosophila*’s track record in studying human viruses and discuss the advantages and limitations of using fruit flies for SARS-CoV-2 research. We also review studies that already used *Drosophila* to investigate SARS-CoV-2 protein pathogenicity and their damaging effects in COVID-19 relevant tissues, as well as studies in which the fly was used as an efficient whole animal drug testing platform for targeted therapeutics against SARS-CoV-2 proteins or their host interacting pathways.

## Introduction

**Fig. 1:**
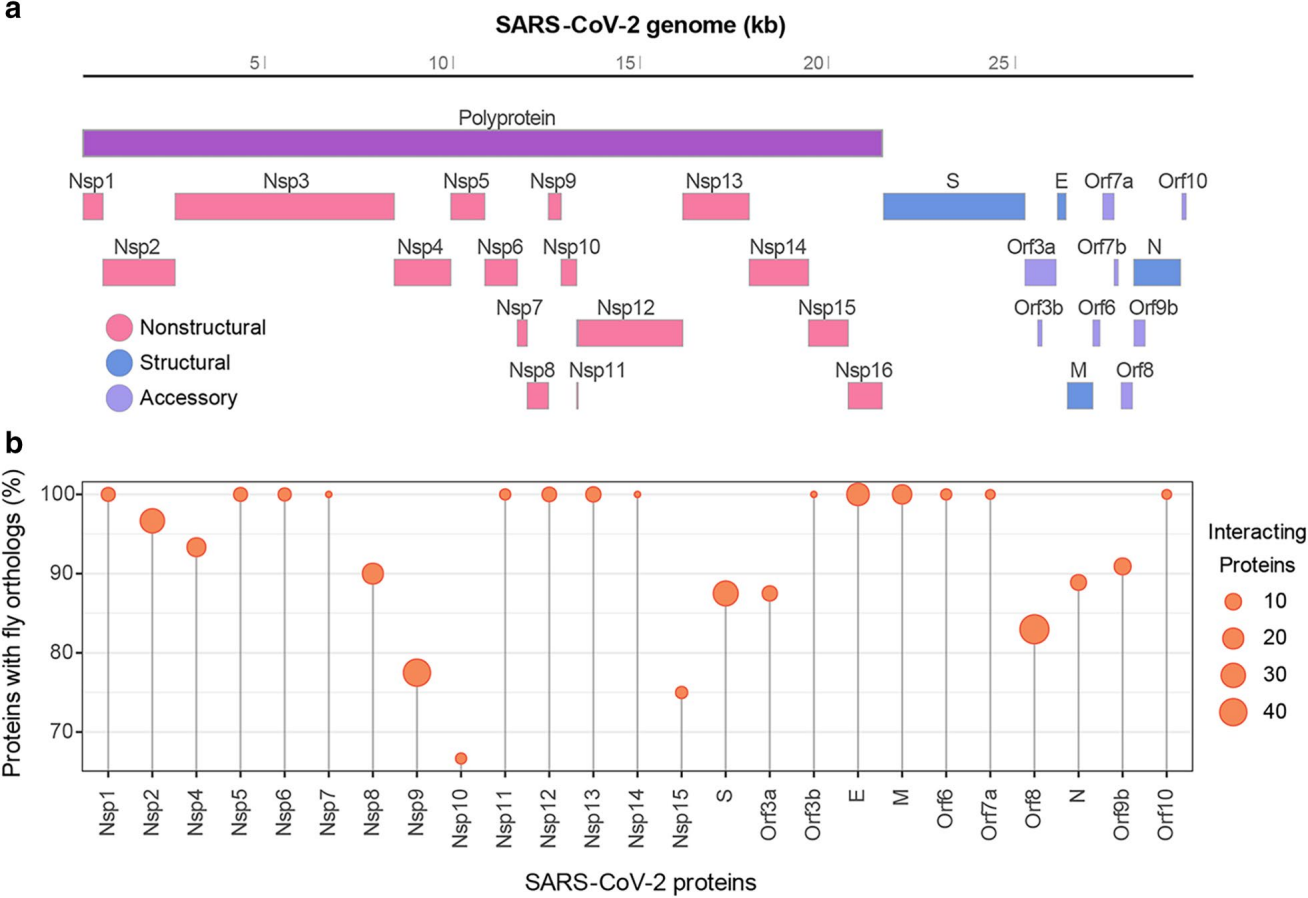
SARS-CoV-2 proteome and conservation of human host interacting proteins in fly.** A** Black bar represents SARS-CoV-2 genome, colored bars below represent the genome-encoded virus proteins. Purple, polyprotein is cleaved through self-protease into non-structural proteins (Nsp) 1–16, shown in pink. The structural virus proteins are depicted in blue: Spike (S), envelope (E), membrane (M) and nucleocapsid (N). The accessory proteins (Orf) are shown in lilac. **B** Lollipop graph displays the percent of human host proteins with fly orthologs (DIOPT ≥ 2; https://www.flyrnai.org/cgi-bin/DRSC_orthologs.pl) for each SARS-CoV-2 protein virus-host network, as reported by Gordon et al. [1]. Size of lollipop top indicates total number of human interacting proteins in network

By every indication, coronaviruses are here to stay. SARS-CoV-2 (severe acute respiratory syndrome coronavirus 2; 2019-present) is the third of highly pathogenic coronaviruses to wreak havoc in the human population within the last two decades, preceded by SARS-CoV (2002–2003) and MERS-CoV (Middle East respiratory syndrome-CoV; 2012-present). Even though these viruses share many clinical features as well as conserved sequences, notable differences have been reported. Coronavirus Orf1ab and Orf1a are mainly conserved, these sequences encode polyproteins that mature into non-structural proteins (Nsp)1–16 through auto-proteolytic cleavage. On the other hand, the genome sequence encoding the accessory factors (Orfs) diverges considerably between the differen coronaviruses [1] (Fig. 1A; SARS-CoV-2). These Orf virus proteins lack well-defined domain structures hampering functional prediction. Their virus-specific attributes strongly imply they facilitate unique host interactions, which in turn could underly the pathogenic differences seen in the clinical presentation of infection with SARS-CoV, MERS-CoV, SARS-CoV-2 and other human coronaviruses. Through tremendous effort and unprecedented data and resource sharing, the global scientific and medical communities have developed multiple vaccines that are currently being administered across the world at a rapid pace under emergency use authorization. This has given us a real chance at fighting SARS-CoV-2 and ending the pandemic. Unfortunately, viruses mutate frequently, already mutations in the SARS-CoV-2 spike protein have been linked to increased virulence through higher infectivity and improved immune evasion potential [2]. In order to prepare for future outbreaks and to protect the human population, we need to gain a deeper understanding of coronavirus protein function, their interacting host proteins, control of host pathways, and how these processes convey pathogenicity at host tissue, organ and systemic levels. This knowledge will greatly benefit treatment strategies (target identification, drug design and development) and management of viral spread.

## SARS-CoV-2 virus-host interactions underlying pathogenicity

Investigations of SARS-CoV-2 virus-host interactions have reported interaction networks specific to each of the SARS-CoV-2 proteins [1, 3]. Through network and Gene Ontology analyses, these studies have revealed host pathways that are likely affected following infection through direct interaction with virus proteins. Around 40% of the interacting host proteins were associated with endomembrane compartments or vesicle trafficking, the remainder were enriched for roles in the innate immune response, host translation machinery and the ubiquitination complex [1]. The latter is quite often targeted by viruses to facilitate replication, and often associated with virulence. Another study generated both intra-virus and virus-host protein interaction networks, these provided insights into the formation of potential virus protein complexes as well as host pathways likely affected through interaction with virus proteins [3]. Further this study undertook global proteome analysis in peripheral blood mononuclear cells (PBMCs) from COVID-19 (coronavirus disease 2019) patients, which revealed upregulated host proteins involved in neutrophil activation and blood coagulation, while mediators of T cell receptor signaling were downregulated. These results have provided clues to the pathomechanism likely leading up to the cytokine storm (hypercytokinemia) found so detrimental in late-stage COVID-19 patients. Based on the known pharmacological profile for various compounds, both research groups proposed inhibitors targeting specific virus-host interactions. The first used their initial study results and extended the data obtained by comparing interaction networks for SARS-CoV, MERS-CoV and SARS-CoV-2 [1, 4]. They proposed, among others: Cell entry, camostat/camostat mesylate, nafamostat/nafamostat mesylate, captopril; Nsp6-SIGMAR1—(hydroxy)chloroquine, chlorpromazine, amodiaquine, amiodarone, tamoxifen and propanalol; Nsp7-PTGES2—indomethacin; Nsp7/Orf9c-NDUFs—metmorfin; Nsp14-IMPDH2—ribavirin; Orf9b-MARK2/3—ruxolitinib; Nsp4/Nsp9/Orf6-NUPs RAE1—selinexor. The second group proposed several IL-6, IL-8 and JAK inhibitors [3], including: IL-6 inhibitor—tocilizumab. All of the compounds listed here are currently in clinical trials to test their efficacy as potential treatments for COVID-19 (clinicaltrials.gov; data obtained March 01, 2021).

Studies defining virus-host protein interaction networks and changes in proteomic profiles to infer host pathways affected by SARS-CoV-2 infection have provided a wealth of information and have led to the identification of potential therapeutic inhibitors. However, of the hundreds of high-confidence SARS-CoV-2 virus-host protein–protein interactions identified in each study, only 45 (~ 16%) were shared [1, 3]. These differences might be explained by the different purification methods and cell lines used in each study, thus additional studies are warranted. Several recent reports have investigated these virus-host interactions in more detail, using in vitro models to identify virus-mediated disrupted pathways and cellular damage [5]. Their findings have highlighted differences between the coronaviruses, singled out the most toxic virus proteins, provided additional insights into host protein targets and underlying pathogenic mechanisms, and identified and tested compounds for therapeutic intervention. However, few of these studies have included primary cells of disease-relevant tissues, and even those are inherently limited as they cannot capture the physiological context of a whole-body system. COVID-19 has been characterized by affecting multiple different organs that can lead to system-wide shut down as disease progresses. In vivo studies will be crucial to delineate the tissue-specific pathogenic effects caused by SARS-CoV-2 infection.

## Current mammalian models for SARS-CoV-2 research

**Fig. 2:**
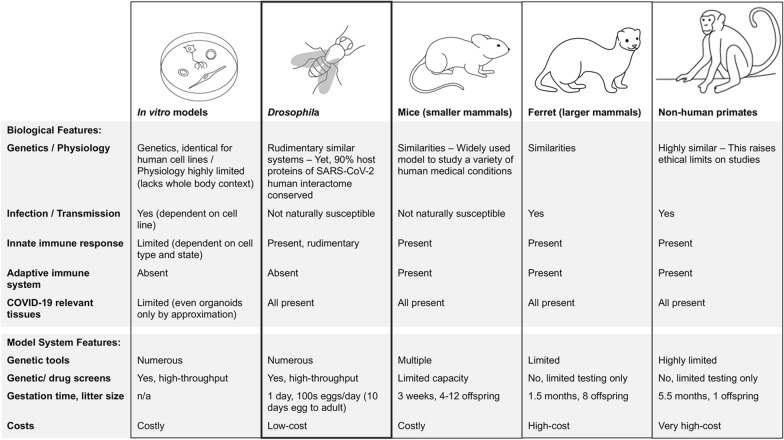
Advantages and limitations of available model systems for SARS-CoV-2 research. Non-human primates, cynomolgous/rhesus macaques

Animal model systems are widely used to investigate the infectious and pathogenic mechanisms underlying viral diseases. Typically, these are mammalian models like mice, hamsters, ferrets and non-human primates, since their genetic and physiological similarities make them well-suited to recapitulated human symptomatology [6–8]. Indeed, transgenic and knock out mice have been used to study the SARS-CoV-2 and host entry factors, host age-associated susceptibility, immune response to infection, for the development of neutralizing antibodies and to determine vaccine efficacy [9–14]. Syrian hamsters and ferrets have uncovered aerosol transmission of SARS-CoV-2 and have been used to study the neutralizing antibody response to infection and infection inhibitory agents [15–17]. Non-human primates, which most closely recapitulate human pathophysiology, have been used to study aging as a factor of COVID-19 severity, re-exposure immune responses, as well as to test the efficacy of therapeutic interventions and vaccine candidates [18–21]. However, limitations such as natural susceptibility (mice transgenic for human ACE2 are required due to limited natural infection), genetic tools available, reproduction times, small progeny, ethical concerns (often increase with the size of the animal) and the high maintenance cost of most of these mammalian model systems, make them unsuitable for genetic (variant) screens, large-scale drug screens or expansive risk factor modeling (Fig. 2). Indeed, to date, the animal studies into SARS-CoV-2 have focused on infection and virus replication. And while extremely valuable, these tell us little about the pathogenic mechanisms that could explain how SARS-CoV-2 proteins are able to hijack host systems and cause damage to so many different tissues.

## Rational for using *Drosophila* to study SARS-CoV-2

The fruit fly *Drosophila* has been developed into a powerful research model platform for the study of a wide variety of human diseases, including several caused by virus infection. This has been made possible by its unique biological and model system features (Fig. 2). Notably, most signaling pathways are not only conserved from fly to human, but many have been first identified and studied in flies [22]. In fact, a remarkable 90% of human proteins in the SARS-CoV-2 human interactome [1] are conserved between flies and humans [23] (Fig. 1B). A separate study, combined the same interactome [1] with one of master regulators, i.e., the parts of the human interactome most affected by SARS-CoV-2 [24] and likewise found high levels of conservation from fly to human based on sequence homology and protein expression data [25]. The compact genome of *Drosophila* carries little redundancy which facilitates data interpretation. Like mice, flies are not naturally susceptible to SARS-CoV-2 and would require transgenic lines to facilitate virus entry. These flies would be designed to express host cell receptors and any additional host factors required for virus entry that have no fly homologs, as well as those factors needed to progress through the virus life cycle thus leading to infection. For these later stages of replication and shedding, the virus heavily depends on host cell systems. These metabolic pathways are highly conserved from fly to human and for several their discovery in fly precedes identification of their mammalian counterparts [22], suggesting the fly might accommodate viral needs once inside its cells. Alternatively, the virus could be adapted to enable cell entry in new species. For example, fly cells lack a sialic acid residue expressed on human host cells that is bound by the influenza virus to facilitate cell entry. Therefore, one study adapted the virus by replacing influenza HA and NA genes with vesicular stomatitis virus (VSV) glycoprotein G [26]. Since VSV can infect both human and fly, the newly generated “Flu-VSV-G” virus was able to enter fly cells. Another option would be to overexpress specific virus genes to study select proteins and their host pathogenic effects. A highly effective strategy as we shall see below, and possibly favorable due to safety concerns when using infective human viruses in the flying fruit flies.

As a whole, the fruit fly is well-equipped to study many aspects of virus-host interactions, pathogenic mechanisms and affected tissues. Although lacking an adaptive immune system, *Drosophila* has an innate immune system with essential components similar to humans, including highly conserved JAK/STAT, Toll receptor, NF-κB and JNK signaling pathways as well as antivirus response factors such as STING [27–29]. For example, Zika virus (ZIKV) infection of the fly brain was shown to induce an NF-κB signaling response which led to activated autophagy in a *Drosophila* stimulator of interferon genes (dSTING)-dependent manner [28]. STING is an innate immune signaling pathway, both the pathway and its autophagy-inducing function are highly conserved from fly to human [28]. Upon infection with dengue virus, fly S2 cells evoke an RNAi response [30]. Using an RNA interference screen 116 candidate host factors for dengue virus replication were identified, of these 42 had human orthologs that similarly promoted virus replication [30]. Infection with West Nile virus has also been shown to induce an RNAi protective response in both *Drosophila* and human cells [31–33]. Vesicular stomatitis virus (VSV) infection is recognized by the fly Toll-7 receptor and its mammalian counterpart TLR7, which bind viral glycoproteins. Thereby invoking an antiviral response, including increased autophagy which inhibits virus replication using an NF-κB-independent process [34, 35]. These studies demonstrate overlap between fly and human antiviral defenses and provide examples of using flies to investigate how viruses affect the host innate immune system response.

Furthermore, *Drosophila* contains a coagulation system with blood clotting components similar to their human counterparts [36]. For example, von Willebrand factor (VWF) is a blood glycoprotein essential in human coagulation with fly ortholog Hemolectin (Hml) [37, 38], and human clotting factor IIIa is represented by fly homolog Transglutaminase (Tg) [39]. Both VWF, Hml and factor IIIa, Tg have been shown to elicit an innate immune response in addition to directly facilitating clotting [39, 40]. Furthermore, factors indirectly supporting coagulation have equivalents in fly such as human Adipocyte Plasma Membrane Associated Protein (APMAP), fly orthologs Hemomucin (Hmu) and Strictosidine synthase-like 2 (Ssl2), and human Lymphocyte Antigen (LY86; a.k.a. MD1), a member of small immunoglobulin (Ig)-like family, with two members of the Ig family (CG11314 and CG11315) released during hemolymph coagulation in fly [39, 41]. Despite the variability in clotting seen even within the insect populations, these commonalities between fly and human coagulation processes open the door to use *Drosophila* to study the effects of SARS-CoV-2 proteins on coagulation and to further explore the link between coagulation and innate immunity. An urgent topic since SARS-CoV-2 infection has been linked to increased blood clotting that causes system-wide damage in patients [42, 43].

Arguably *Drosophila*’s greatest potential lies in studying the metabolic pathways disregulated by virus proteins. The components and functions of metabolic pathways are highly conserved from fly to human [22]. Thus, flies could be used to investigate the metabolic systems hijacked by SARS-CoV-2 to promote virus replication. In addition, several metabolic-related risk factors, such as diabetes and obesity, have been associated with more severe COVID-19 progression [44, 45]. The fly has already proven to be a valuable model in studying human diet, obesity, diabetes and metabolic diseases [46–48]. These existing models could be readily employed to study COVID-19 risk factors. Moreover, *Drosophila* has all the major organs affected in COVID-19, including heart, lung, muscle, kidney, blood and brain. Despite its small size, the fly captures many biological features relevant to humans in general and SARS-CoV-2 infection specifically. Even though the morphology and physiology of these organs are different from humans, the similarities at the cellular and genetic levels are striking [49, 50]. What makes *Drosophila* such a powerful and effective model is that it offers in vivo data with the speed and cost of a cell culture system. Mammalian models resemble human genetics and physiology to a greater extent than the fruit fly, however this also raises ethical issues. Moreover, the larger the mammal, the longer the gestation time, smaller the number of progeny and more labor intensive and costly the research (Fig. 2). Speed is of the essence when responding to a virus outbreak, and *Drosophila*’s ease-of-use, cost-efficiency and plethora of genetic tools are unrivaled. And, unlike its mammalian counterparts, the fly is capable of large in vivo genetic and pharmacological screens. Taken together, when it comes to functional studies of virus-host interactions, *Drosophila* offers astonishing advantages and provides a powerful model system capable to instantly adapt and respond to anything coronaviruses throw at us.

## Track record of using *Drosophila* to study human viruses

Even though generally underutilized in viral studies, despite its potential, the fruit fly has been successfully used to investigate the molecular and physiological aspects of human virus-induced pathogenic effects on host cells in recent years, including human immunodeficiency virus (HIV), ZIKV, dengue, West Nile, as well as VSV, Epstein-Barr, human cytomegalovirus, and SARS-CoV [51–53]. For example, *Drosophila* has been employed to investigate influenza A virus, these studies have uncovered novel insights into influenza pathogenesis [54]. Transgenic fly lines that expressed influenza M2 protein in the wing and eye showed defects in both tissues with more severe phenotypes in males compared to females [55]. Moreover, when treating these flies with amantadine, an antiviral agent, the M2 phenotype was effectively diminished, demonstrating the flies’ potential and value in pharmaceutical screens. Next, the flies were screened for mutations in host interacting genes, which revealed several host modifiers of the influenza M2-mediated phenotype. Other studies have made use of *Drosophila*’s screening capabilities to identify RNA silencing suppressors of influenza phenotypes mediated by different NS1 variants [56]. Several of these findings have been subsequently confirmed in mammalian cells and mice. A major influenza study carried out a genome-wide RNAi screen in fly to identify virus-host interactions and uncovered previously unrecognized host genes that turned out to be essential for influenza replication [26]. In the above examples, the RNAi screens were carried out in fly cell culture systems, however we and others have previously applied similar screens to live flies. For example, we used an RNAi screen to validate candidate human genetic variants associated with congenital heart disease, which revealed the importance of histone H3K4 modifying proteins in heart development [57]. These same techniques could be rapidly applied to study new viral strains and host genetic variants that might alter risk of severe disease progression or could influence effectiveness of pharmacological interventions.

In another example, the fly *UAS*-*Gal4* gene expression system was applied to study SARS-CoV Orf3a [58] and M (membrane) proteins [59]. Overexpression of SARS-CoV Orf3a caused cytotoxicity in the *Drosophila* eye, and Orf3a protein cellular localization was shown to be comparable in fly and human VeroE6 cells. Using a forward genetic screen in fly the authors identified host modifier genes of 3a protein pathogenicity and found nearly 60% of these have human orthologs that are expressed in the lung. These data have implicated trafficking and clathrin-mediated endocytosis, altered gene regulation, as well as host modifiers of SARS-CoV 3a protein-mediated apoptosis. Overexpression of SARS-CoV M protein caused apoptosis in the fly eye, which was attenuated by overexpressing apo-cytochrome *c* protein. Further assays demonstrated a role for several caspases in M protein induced apoptosis. A forward genetic modifier screen revealed PDK-1 kinase, an upstream effector in the Akt pro-survival signaling pathway, could attenuate SARS-CoV M protein-mediated pathogenesis. Notably, the human ortholog of PDK-1 kinase is expressed in the lung. Findings from both reports have been replicated and subsequently expanded upon [60] using human in vitro systems, indicating conservation of the virus-host interactions between flies and humans, an important attribute for an animal model of human disease.

## *Drosophila* in SARS-CoV-2 research, to date

Despite the fruit fly’s relative limited use to study human viruses and SARS-CoV-2 having arisen just over a year ago, two fly studies have been reported. The bioRxiv preprint server lists one article that used *Drosophila* to investigate SARS-CoV-2 [61]. The study focuses on SARS-CoV-2 Orf3a, using the *UAS-Gal4* system with tissue-specific drivers, the viral protein was overexpressed in the fly central nervous system (*elav-Gal4*), photoreceptors (*GMR-Gal4*), and striated and smooth muscle (*Mef2-Gal4*). Flies with Orf3a overexpression in muscle showed no detectable effect on longevity, nor changes in motor function or muscle patterning. All flies with photoreceptor Orf3a overexpression showed a rough eye phenotype, indicative of a patterning defect and apoptosis. In contrast to these mild Or3fa-mediated phenotypes, expression in the central nervous system caused partial larval lethality and reduced lifespan, combined with impaired motor function (muscle weakness) and marked abdominal swelling [61]. At the molecular level, Orf3a induced Caspase-3 cleavage as well as increased expression levels of Toll pathway and immune deficiency (IMD) pathway proteins which are markers of the immune inflammatory response. These findings support previous observations from in vitro (Vero E6 cells) studies and COVID-19 patients. Chloroquine phosphate has been proposed to mitigate some of the pathway disruptions caused by Orf3a and is currently in clinical trials for treatment of COVID-19 (https://clinicaltrials.gov). Treating the flies with chloroquine phosphate attenuated the SARS-CoV-2 Orf3a-induced phenotypes significantly. In addition, it reduced the Orf3a-mediated Caspase-3 cleavage as well as reduced Toll pathway activity, indicating the molecular pathways of pharmacological intervention. Clinical trials have found no effect of chloroquine phosphate on improving COVID-19 patient survival, however, the authors argue that the treatment might benefit patient long-term post viral symptomatology.

A recent publication from our group surveyed 12 SARS-CoV-2 proteins in flies, prioritized by their predicted likelihood to instigate pathogenic host interactions [23]. Similar to the study above, we studied the proteins in multiple tissues, in this case muscle and trachea (fly equivalent of the lung); both tissues are affected in COVID-19. Notably, the SARS-CoV-2 genes found to convey highest pathogenicity (i.e., *Nsp6*, *Orf6* and *Orf7a*) in the in vitro human cell culture (HEK 293 T) model were also most pathogenic in our in vivo transgenic flies system [23, 62]. In our hands, system-wide overexpression of protein SARS-CoV-2 Orf3a (*Tub-Gal4*) reduced overall lifespan [23] but did not increase early lethality (death prior to eclosion) as observed when SARS-CoV-2 Orf3a was overexpressed in the fly central nervous system (*elav-Gal4*) [61]. In line with previous studies of human viruses in fly, orthology analysis revealed astounding levels (90%) of conservation for the SARS-CoV-2 virus-host interactome between humans and flies [23] (Fig. 1B). Flies transgenic for Nsp6, Orf6 or Orf7a proteins, each displayed muscle deficits with dramatically reduced mitochondria, as well as significantly reduced tracheal (lung) branching [23]. These findings are in line with clinical observations, which report COVID-19 is a respiratory disease, with muscle weakness among the persisting symptoms [63]. Both SARS-CoV and SARS-CoV-2 Orf6 proteins have been shown to act as antagonists of IFN signaling [64]. In addition, SARS-CoV-2 Orf6 has been implicated in direct interaction with the host NUP98-RAE1 complex at the nuclear pore [1, 3, 62, 65]. Notably, these nuclear export proteins are extremely highly conserved from fly to human [23]. Moreover, treatment with selinexor, an FDA-approved inhibitor of nuclear export, attenuated cytotoxicity in our human in vitro model, as well as the Orf6-mediated tracheal (lung) branching and muscle-mitochondria phenotypes observed in our in vivo fly model [23, 62]. This efficacy was specific to SARS-CoV-2 Orf6, as selinexor was unable to diminish the Nsp6 or Orf7a induced phenotypes. These findings suggest that despite phenotypic overlap the underlying pathogenic mechanisms are specific to each virus protein. Of note, two clinical trials have been registered for selinexor (KPT-330) (https://clinicaltrials.gov), which aim to evaluate its effect on outcomes of clinical recovery in patients with active COVID-19 infection (NCT04349098 in the general population, has been completed, but results have not yet been reported; NTC04534725 in the cancer population, study is currently recruiting). It will be interesting to see how their findings compare to those in the fly model. Combined, these data strongly convey the human relevance and potential of *Drosophila* as an in vivo model system to identify tissue-specific pathogenic effects caused by virus-host protein interactions and for testing pharmacological agents targeting to disrupt these interactions.

## Future perspective and conclusion

Taken together, the existing literature has established *Drosophila* as a promising model organism in studies of virus-caused human disease. Fly is the furthest removed from human in terms of genetics and physiology, often possessing more rudimentary versions of specific signaling systems. However, the pathways disrupted by virus proteins following infection appear to be highly conserved from fly to human (90% for SARS-CoV-2 human interactome). The human relevance of findings made in fly have already been demonstrated in reports that used *Drosophila* to study various human viruses and pathological pathways. Moreover, the fly’s strength lies in providing the benefits of an in vivo animal model (whole body context, tissue-specific pathogenic investigations), while also offering the speed and multitude of genetic tools of an in vitro system. Therefore, we foresee the fly to be a powerful ally in combatting future outbreaks. *Drosophila* provides a means to not only prepare, but also to respond immediately to any newly emerging virus strains. The benefit of which is all too clear with several mutant SARS-CoV-2 strains rapidly taking over in the ongoing pandemic and already putting a strain on the available vaccines. The fly can be easily used to investigate all of the virus proteins individually or adapted to carry multiple transgenes to research the pathogenic effects of known virus-self or virus-host complexes. Moreover, extensive genetic modifier screens can be used to reveal host pathways contributing to pathogenicity and candidate targets for pharmacological intervention. These studies could be further expanded to screen virus and host genetic variants to prioritize those more likely to increase pathogenicity and thereby pose a risk to population/patient health. Finally, the first SARS-CoV-2 fly studies have already demonstrated their pharmacological application. Indeed, flies can be used in fast, in vivo, extensive screens for pharmacological interventions. Altogether, we propose to use *Drosophila* in powerful initial screens to identify promising candidates, which can then be investigated further in the much more labor intensive and costly studies using mammalian model systems.

In conclusion, *Drosophila* is a versatile, adaptable and cost-effective model with an unmatched array of tools, that can provide a fast response to match virus mutation pace. We foresee its value as a large-scale in vivo screening tool to prioritize candidates (genetic and pharmacological) for subsequent extensive study in mammalian model systems. Rest us to say: Do not underestimate the power of the mighty fruit fly, which could be an astonishing ally in the biological arms race against coronaviruses.

## Data Availability

All data and materials generated in this study are available publicly upon request.

## Abbreviations

ACE2: Angiontensin I converting enzyme 2
APMAP: Adipocyte plasma membrane associated protein
COVID-19: Coronavirus disease 2019
DIOPT: DRSC Integrative Ortholog Prediction Tool (https://www.flyrnai.org/cgi-bin/DRSC_orthologs.pl)
E protein: Envelope protein
*elav*: Embryonic lethal abnormal vision (central nervous system-specific expression)
FDA: Food and Drug Administration (USA)
*GMR*: Glass multiple reporter (pan-retinal-specific expression)
H3K4: Histone H3 lysine K4
HEK 293T: Human embryonic kidney cell lines
HIV: Human immunodeficiency virus
Hml: Hemolectin
Hmu: Hemomucin
IFN: Interferon
Ig: Immunoglobulin
IL-6, IL-8: Interleukin-6, -8
IMD: Immune deficiency pathway
IMPDH2: Inosine-5′-monophosphate dehydrogenase 2
JAK/STAT: Janus kinase/signal transducer and activator of transcription protein
JNK: C-Jun N-terminal kinase
M protein: Membrane protein
MARK2/3: Microtubule affinity regulating kinase 2/3
*Mef2*: Myocyte enhancer factor 2 (muscle-specific expression)
MERS-CoV: Middle East respiratory syndrome coronavirus
NDUFs: NADH-ubiquinone oxidoreductase subunits
NF-κB: Nuclear factor kappa-light-chain-enhancer of activated B cells
NUPs: Nuclear pore proteins (e.g. NUP98)
N protein: Nucleocapsid protein
Nsp: Non-structural protein
Orf: Accessory protein
PBMCs: Peripheral blood mononuclear cells
PDK-1: Phosphoinsitide-dependent kinase 1
PTGES2: Prostaglandin E synthase 2
RAE1: Ribonucleic acid export 1
RdRp: RNA-dependent RNA polymerase
RNAi: RNA interference
S protein: Spike protein
SARS-CoV(-2): Severe acute respiratory syndrome coronavirus
SIGMAR1: Sigma non-opioid intracellular receptor 1
Ssl2: Strictosidine synthase-like 2
(d)STING: (*Drosophila*) Stimulator of interferon genes
Tg: Transglutaminase
TLR7: Toll-like receptor 7
*Tub*: Tubulin (ubiquitous expression)
*UAS-Gal4*: *UAS* Enhancer sequence, *Gal4* transcriptional activator
Vero E6: African green monkey kidney cell line
VSV: Vesicular stomatitis virus
VWF: Von Willebrand factor
ZIKV: Zika virus
